# Metabolic Disorder in Chronic Obstructive Pulmonary Disease (COPD) Patients: Towards a Personalized Approach Using Marine Drug Derivatives

**DOI:** 10.3390/md15030081

**Published:** 2017-03-20

**Authors:** Palma Lamonaca, Giulia Prinzi, Aliaksei Kisialiou, Vittorio Cardaci, Massimo Fini, Patrizia Russo

**Affiliations:** 1 Clinical and Molecular Epidemiology, IRCSS San Raffaele Pisana, Via di Valcannuta 247, I-00166 Rome, Italy; palma.lamonica@sanraffaele.it (P.L.); giulia.prinzi@sanraffaele.it (G.P.); alesseus@gmail.com (A.K.); 2 Department of Pulmonary Rehabilitation, IRCCS San Raffaele Pisana, Via della Pisana 235, I-00163 Rome, Italy; vittorio.cardaci@sanraffaele.it; 3 Scientific Direction, IRCSS San Raffaele Pisana, Via di Valcannuta 247, I-00166 Rome, Italy; massimo.fini@sanraffaele.it

**Keywords:** chronic obstructive pulmonary disease, comorbidities, management strategy, marine compound, metabolic disorder, inflammation, systems approaches

## Abstract

Metabolic disorder has been frequently observed in chronic obstructive pulmonary disease (COPD) patients. However, the exact correlation between obesity, which is a complex metabolic disorder, and COPD remains controversial. The current study summarizes a variety of drugs from marine sources that have anti-obesity effects and proposed potential mechanisms by which lung function can be modulated with the anti-obesity activity. Considering the similar mechanism, such as inflammation, shared between obesity and COPD, the study suggests that marine derivatives that act on the adipose tissues to reduce inflammation may provide beneficial therapeutic effects in COPD subjects with high body mass index (BMI).

## 1. Introduction

**Figure 1:**
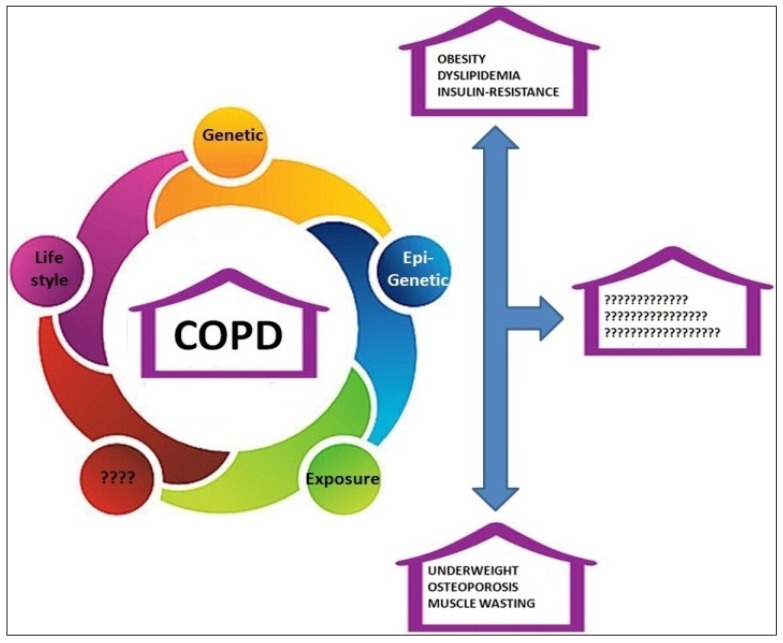
Constellations of different Metabolic Disorders and theoretical underlying “types” in COPD. Distinct pathophysiological mechanisms (genetic, epigenetic, environmental factors, life style) might underlie the occurrence of groups of Metabolic Disorders in patients with COPD.

Chronic Obstructive Pulmonary Disease (COPD) is a complex illness whose development depends on the interaction between environmental and genetic risk factors. COPD is characterized by enduring airflow constraint, often resistant to bronchodilators and corticosteroids [1,2,3,4]. Although, COPD is considered “*part of a worldwide tobacco-related disease epidemic*”, at least one fourth of patients are non-smokers [4]. Thus, other environmental factors such as indoor/air pollution, second-hand smoke (during pregnancy, early childhood, or life), chemical fumes or dust as well as genetic factors may contribute to its development [1,2,3,4]. Several regions of the genome may be associated with COPD. Genetic variants in the alpha-1 antitrypsin (AAT, autosomal recessive) gene, discovered in the early 1960s, are associated with a major risk to develop COPD. The onset of COPD is between 40 and 50 years in smoker carriers Alpha-1 Antitrypsin Deficiency (AATD), whereas, in non-smokers, the onset is delayed to 60 years [5]. However, these variants account for only 1%–2% of all COPD cases [5]. Genetic variations in the cluster on chromosome 15, encoding the nicotinic acetylcholine receptor subunits (CHRNA5-CHRNA3-CHRNB4), are correlated with tobacco addiction and increased risk of COPD, peripheral artery disease, lung cancer and obesity [6,7,8]. Different studies on lung tissue and peripheral blood identified increased expression of genes related to inflammatory pathways and immune regulation [9]. More studies are warranted to recognize those genes and pathways occurring in COPD. Pathological signs such as remodeling and straitening of the small airways anddestruction of the lung parenchyma are consequential to the chronic inflammation. Chronic inflammation contributes to the extra pulmonary effects, the so-called “systemic effects”, of COPD and regulates disease expression, burden, and mortality [10,11]. COPD patients commonly show several progressive failures connected to the “*cardiopulmonary-metabolic axis*” (CMA). The CMA provides oxygen and nutrients to the body. A link between metabolic syndrome (MetS code E88.81), or better to metabolic disorder (MetD), and lung diseases has been reported by several cross-sectional and longitudinal studies (as reviewed, recently, by Baffi et al. [12]). The concept of MetS evolved over time to MetD; indeed, MetD is a complex disorder thought as a cluster of conditions sustaining a “*disorder in energy use and storage*” characterized by central obesity, dyslipidemia, high blood sugar levels (hyperglycemia), hypertriglyceridemia, and low high-density lipoprotein cholesterol levels. MetD is also associated with a prothrombotic and a pro-inflammatory state [13]. A recent systematic review, that includes 19 studies involving 4208 COPD patients, evidences the presence of MetD in the 34% of population with high prevalence of arterial hypertension, abdominal obesity, and hyperglycemia [14]. In a group of elderly 877 patients (74 years median) admitted to San Raffaele Group Pulmonary Rehabilitation Units, 84% patients have 1–4 comorbidities and 10% >4 with an increasing trend of multimorbidity over recent years, mainly 58.6% (514 patients) suffer from arterial hypertension, 24.4% (214 patients) diabetes, 21.4% (188 patients) obesity and 7% (61 patients) dyslipidemia [15]. It has been reported that one or more components of the MetD are present in each patients that are in part steroids treatment and/or physical inactivity independent [13]. Actually, although the impacts of MetD, as well as other comorbidities, in the management of COPD is well acknowledged, no certainty exists on treatment of MetD to reduce its effects on the respiratory system. The uncertainty is linked to the complex interplay among genes, epigenetic, lifestyle and environmental exposures that lead to different COPD phenotypes (i.e., obesity/dyslipidemia/insulin-resistance or underweight/osteoporosis/muscle wasting, see Figure 1).

The sub-optimal phenotyping of COPD patients partially explains the nonoccurrence of therapeutic COPD breakthroughs. Treatment may be thoughtfully palliative and moreover different patients respond in different ways to treatment. Commonly, the presence of MetD does not change the treatment of COPD that is treated independently of it [16]. 

**Figure 2:**
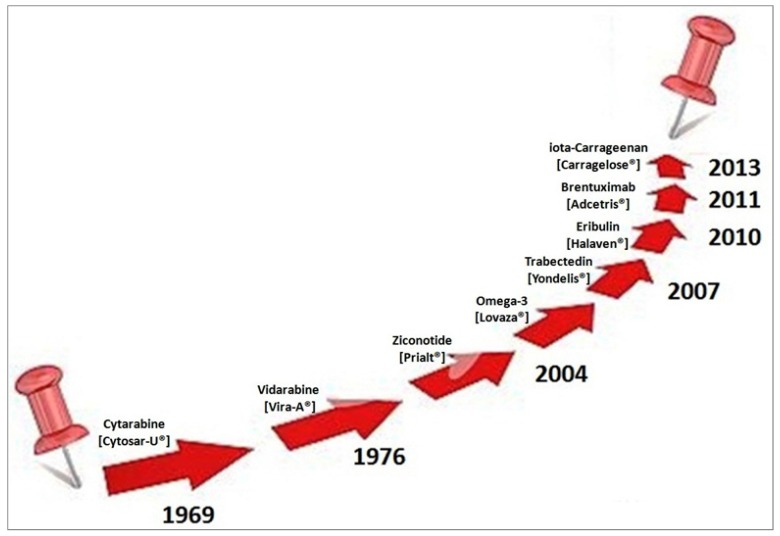
Marine drugs approved by FDA (U.S. Food and Drug Administration)/EMA (European Medicines Agency) from 1969 to 2013.

Natural products, obtained from living organisms, such as most plants, microbes, and animals are an incomparable source of molecular diversity in drug discovery and have been generated new drugs (secondary metabolites) or drug derivatives [17,18]. Starting from 1969, U.S. Food and Drug Administration (FDA)/European Medicines Agency (EMA) approved eight drugs obtained from marine sources (Figure 2 and Table 1). 

Lovaza^®^, obtained by fish oil, is anΩ-3-acid ethyl esters (ethyl esters of eicosapentaenoic acid (EPA) and docosahexaenoic acid (DHA)), authorized as an adjunct to diet to reduce triglyceride (TG) levels in adult patients with severe (≥500 mg/dL) hypertriglyceride (HTG) [19]. Different mechanisms of action have been proposed for Ω-3-acid ethyl esters including inhibition of diacylglycerol acyltransferase, increased plasma lipoprotein lipase activity, decreased hepatic lipogenesis, and increased hepatic β-oxidation [19]. 

This review describes various compounds of natural marine sources able to modulate several different anti-obesity targets. With the exception of fish oil drug derivatives approved by FDA/EMA all the compounds are in preclinical setting (cell and animal models) and need further experiments to make clear the mechanism of action, the possible side effects and the safety. A possible link between anti-obesity activity and lung function modification is hypothesized. Compounds from marine organisms containing EPA and DHA are not described in detail in this review since there is a copious literature see the recent reviews [19,20,21,22,23].

## 2. COPD and Obesity

Traditionally, COPD is associated with sarcopenia (a condition characterized by loss of skeletal muscle mass and function, frequently occurring in advance age) [24]. However, obesity is currently frequent in COPD, contributing to respiratory symptoms [25] and potentiating several known associated comorbidities such as cardiovascular disease (CVD), type2-diabetes (T2DM), skeletal muscle dysfunction, and obstructive sleep apnea [26]. The occurrence of COPD with sarcopenia and concurrent obesity (i.e., Sarcopenic Obesity: SO) has recently been reported [27]. A study that evaluated 2000 patients with COPD shows that the presence of SO induces worse physical performance and higher systemic inflammatory burden than in patients with COPD alone [27]. The concomitant presence of COPD and obesity is rising at a remarkable rate in the Western world [28]. In these patients, the respiratory symptoms have the tendency to be worst, the daily activities are more restricted and the quality of life (QoL) is poorer than in non-obese subjects. A recent study, examined 3631 COPD patients with a confirmed post-bronchodilator FEV_1_ < 80% predicted and a Body Mass Index (BMI) ≥ 18.5 kg/m^2^ to test the hypothesis of the association between obesity and worse outcomes in COPD. In the above study, the association between obesity and worse outcomes was independent of the presence of comorbidities and was associated, in a dose-dependent manner, with worse QoL, dyspnea, endurance to six minutes walking distance (6 MWD) and severe acute exacerbation of COPD (AECOPD). These associations were strengthened when obesity was analyzed as a dose-dependent response [29]. On the other hand, in a not fully understood reason, obesity seems associated with a reduced mortality risk in COPD patients (so-called “COPD–obesity paradox”) [30]. A recent study, planned to explain the “COPD–obesity paradox”, assessed the causal role of high BMI in COPD exacerbations and pneumonias [31]. Authors analyzed the genetic variants that cause lifelong high BMI in FTO (fat mass and obesity-associated gene, rs9939609), MC4R (Melanocortin-4 Receptor gene, rs17782313) and TMEM18 (transmembrane protein 18, rs6548238) genes to evaluate the consequences of the obesity in COPD patients. The study evaluates a large cohort population of the Copenhagen General Population Study, including 10,883 subjects who had spirometric (FEV_1_ measurement) COPD. The study concludes that genetically determined high BMI is associated with an increased risk of recurrent exacerbations and pneumonias in individuals with COPD, while this was not the case for observationally determined high BMI [31]. 

It has been suggested that adipose tissue may talk to other organs through endocrine functions; specifically, the crosstalk between adipose tissue and lung may be mediated by adipokines [32]. Thus, the serum levels of adipokines are elevated in patients affected by COPD, independently of their smoking habit, and positively correlate with disease severity and ratio of exacerbation [33,34]. Moreover, high adiponectin levels and low leptin/adiponectin ratio are associated with annual forced expiratory volume in 1 s (FEV_1_) decline [33].

Adiponectin plays a key role in carbohydrate and fat metabolism. It is a polypeptide of 30 kDa, expressed and released exclusively from adipocytes of white adipose tissue. Adiponectin acts throughout two principal receptors, AdipoR1 and AdipoR2, activating or inhibiting down-stream signaling pathways such as AMPK and ceramidase (activation) or phosphatidylinositol 3-kinase; wing-less type protein (Wnt)/β-catenin, ERK1/2; nicotinamide adenine dinucleotide phosphate oxidase, STAT3; and nuclear factor κB (NFκB) (inhibition). Adiponectins secretion is stimulated by insulin and inhibited by TNF and IL-6. Secretion of the hormone decreases in the case of obesity [35,36]. Leptin regulates fat mass, food intake, and thermogenesis enhancing the production of TNF and IL-6. It also promotes the production of reactive oxygen species (ROS), and stimulates monocytes proliferation and migration [37]. 

**Figure 3:**
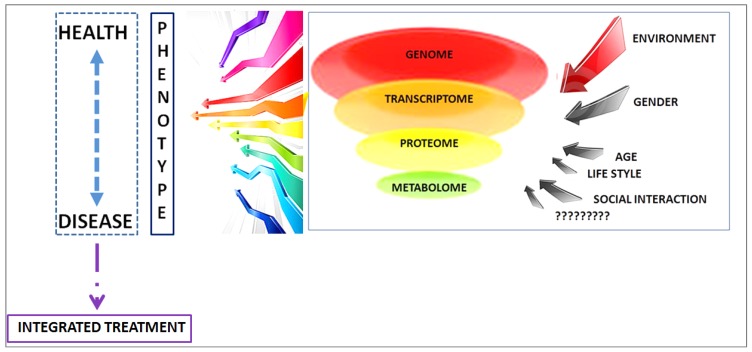
Interaction between omics and environment to determine phenotype health/disease.

Obesity is a complex metabolic disorder in which interactions among genetic/epigenetic, behavioral and environmental factors lead to its development. The World Health Organization (WHO) defines obesity as: “*BMI equal to or greater than 30 kg/m^2^*” [38]. Phenotypically, accumulation of fat in different body regions (principally abdominal) characterizes obesity, while, at the cellular level, obesity implies both an increase in the adipocyte cell size (hypertrophy) and an increase in the adipocyte cell number (hyperplasia) [39]. Actually, diet plays a major role in the probability to develop a chronic disease (better called as non-communicable diseases (NCDs)), especially in the presence of unhealthy habits or overweight. Obesity is a well-known risk factor of increased premature mortality and cardiovascular disease, diabetes, and cancer [40]. The worldwide incidence of obesity doubled over the past decades, leading to increasing rates of diabetes mellitus, cardiovascular disease, and their complications. For adults, WHO defines overweight and obesity as follows: overweight is a BMI greater than or equal to 25; while obesity is a BMI greater than or equal to 30 [41]. According to the different class of obesity, people with class 1 (BMI ≥ 30 and ≤ 35) do not have elevated healthcare costs, but for people in the range of class 2 and 3 (BMI ≥ 35) the healthcare costs rise rapidly in parallel with BMI. The major expenditure is strictly linked to concomitant diseases. It has been estimated that a 5% reduction in weight allows a savings in annual medical care of $US2137 for those subjects with a starting BMI of 40, of $US528 for those of 35, and $US69 for those of 30 [42]. The extensive application of omics-based technologies allows establishing what factors may influence health status, disease development, and an individual’s response to interventions. Metabolomics, that measures the complete balance of metabolites, may be particularly influential in this respect. Indeed, the notion of a personal metabolic phenotype or “metabotype” was coined with the introduction of metabolomics-based research [43,44]. Metabolomics may identify metabolite profiles and biological pathways associated with diet-related diseases [45] and may help in unraveling the relationships between health and disease status (Figure 3), thus among metabolism, obesity and progression of COPD. At the same time, metabolomics may have potential as a clinical tool in risk evaluation and monitoring of disease [46].

## 3. Treatment of Obesity

Until now, no safe and effective anti-obesity drugs that include suppressing appetite, drugs increasing insulin sensitivity, drugs targeting sodium/glucose cotransporters and drugs decreasing lipid absorption have been developed. The FDA, in approving an anti-obesity drug, requires a weight reduction of at least 5% for at least one year (difference between the drug and the placebo groups) in the 35% subjects in treatment [47,48]. The newest drug Saxenda^®^ (liraglutide, rDNA origin) [49,50] is approved by FDA/EMA as “*a treatment option for chronic weight management in addition to a reduced-calorie diet and physical activity to subject who also have one or more complications related to their weight, such as type 2 diabetes, high blood pressure, high cholesterol or obstructive sleep apnea*”. Saxenda^®^ is a glucagon-like peptide-1 (GLP-1) receptor agonist. EMA recommended that whenever a patient does not lose 5% of their initial body weight after 13 weeks, the treatment with Saxenda^®^ should be stopped [50]. As a general observation, every success on pharmacotherapy of weight management is not related with any changes to the obesity cellular/molecular mechanisms and is transitory. The success of pharmacotherapy depends on personalizing treatment considering genetics, behaviors and comorbidities and consequently drug interactions, contraindications, and risk of potential adverse effects.

### Marine Drugs and Obesity and COPD

All over the world, natural products have been the source of food and medicine. Nowadays, ocean habitats are the newest frontier in drug medical research. Since 1969, FDA/EMA have approved eight drugs of marine origin (Figure 2) including important anticancer agents. Although the actualization of this area of scientific exploration is relatively new, the first testimony of marine medicine comes from 2953 BC during emperor Fu His in China as a tax for profits of fish-derived medicine [51]. Indeed, fish oils are marine-derived products that have been in use for millennia. Actually, several reviews on this topic have been published [52,53,54,55,56,57,58]. Different marines organisms (from fishes such as salmon and herring, to krill and squid) contain so-called “marine ω-3 fatty acids” and specifically eicosapentaenoic acid (EPA; 20:5*n*-3), docosapentaenoic acid (DPA; 22:5*n*-3) and docosahexaenoic acid (DHA; 22:6*n*-3) [59]. Both EPA and DHE control metabolism and functions of adipose tissue, supporting the oxidative metabolism via mitochondrial biogenesis and fatty acid oxidation. EPA and DHA also regulate adipocyte glucose utilization and insulin sensitivity (Akt phosphorylation) through Peroxisome proliferator-activated receptor gamma (PPARγ) and 5′ adenosine monophosphate-activated protein kinase (AMPK) activation. EPA and DHA regulating the production of pro-inflammatory chemokines and cytokines may reduce inflammation. In 2001 and 2004, Lovaza^®^/Omacor^®^, a drug containing EPA and DHE, was approved by EMA and FDA [60], respectively, as a lipid-regulating agent. Presently, an ancillary study (R01HL101932 supported by the National Heart Lung and Blood Institute (NHLBI)) is ongoing on a subset of participants in Vitamin D and Omega-3 Hypertension Trial (VITAL Hypertension) (VITAL; NCT 01169259 a five-year U.S.-wide randomized, double-blind, placebo-controlled) with the aim to examine whether marine ω-3 fatty acids (Omacor^®^ 1 g/day) improves respiratory symptoms or reduces the risk of lung infections or reduces the decline of pulmonary function [61]. The VITAL-subcohort was initially composed of 2027 participants of both gender, 50 years and older (adult, senior), from 11 continental U.S. locations. In total, 1973 subjects were randomized and 1924 had lung function tests of acceptable quality, among these 27.3% had mild or moderate Global Initiative for Chronic Obstructive Lung Disease (GOLD) COPD stages and 5.9% had PRISm (Preserved Ratio Impaired Spirometry or “restrictive” spirometry) [61]. Interestingly, the mean BMI of the subjects entering on the study was 29.9 (>30 = obesity) suggesting overweight or obesity.

EPA and DHA control adipose tissue metabolism and functions acting on:

- Adipocyte fat storage and mobilization;
- Adipocyte oxidative metabolism through the stimulation of mitochondrial biogenesis and fatty acid oxidation;
- Adipocyte glucose utilization and insulin sensitivity (Akt phosphorylation);
- Secretion of adipokines; and
- Mitigation of adipose tissue inflammation through production of pro-inflammatory chemokines/cytokines, reduction of M1 macrophage infiltration/-6 derived pro-inflammatory lipid mediators production, being substrates for the formation of some specialized pro-resolving lipid mediator (SPMs), namely resolvins, protectins, and maresins.

**Figure 4:**
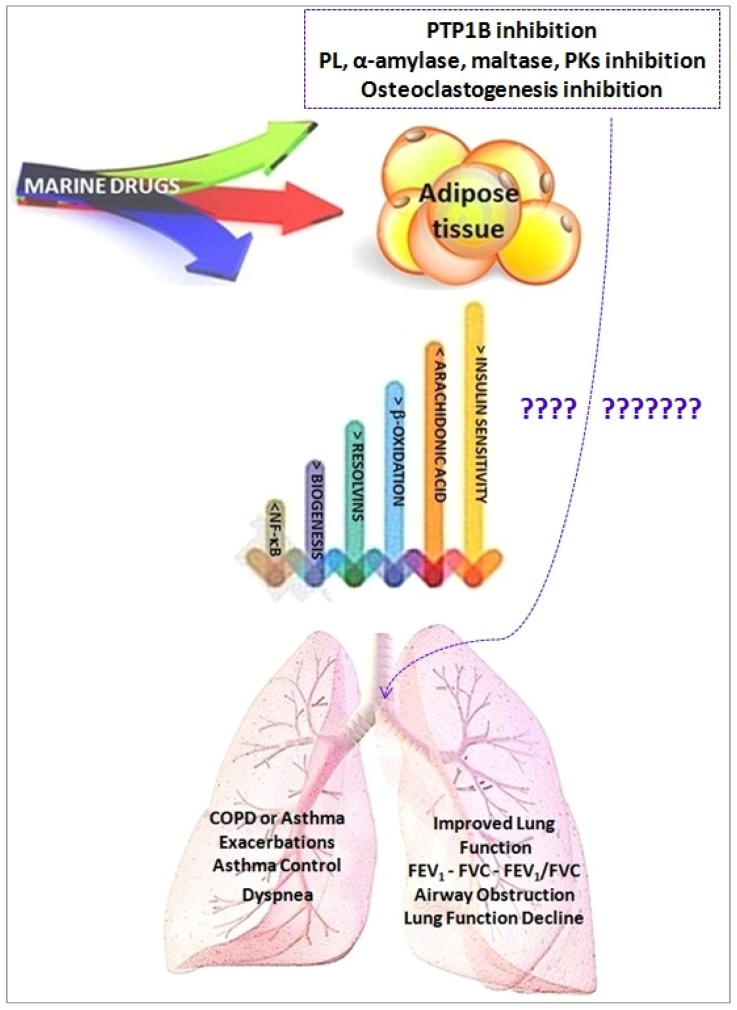
Relationship between adipose tissue and lung function after DHA/EPA administration. Adapted from [23,61]. Protein tyrosine phosphatase 1B (PTP1B); Pancreatic lipase (PL); Protein kinases (PKs).

SPMs, acting throughout specific 7-transmembrane G-protein coupled receptors, take action on neutrophil trafficking, promote macrophage phagocytosis, and block pro-inflammatory cytokine and chemokine production. In human, 15-lipoxygenase produces protectins and resolvins D while, resolvin E series are produced via the acetylated cyclooxygenase-2 or cytochrome P450 pathway. The levels of SPMs in COPD are lower than in non-affected patients suggesting that a scarce activity of SPMs may maintain the status of chronic inflammation and is a cause of the pathobiology of COPD [23]. Figure 4 summarizes possible mechanisms linking the effects of EPA/DHA on adipose tissues and on lung functions. 

Table 2 reports new drugs and drug derivatives obtained by different marine organisms proposed in anti-obesity treatment [62,63,64,65,66,67,68,69,70,71,72,73,74,75,76,77,78,79,80,81,82,83,84,85,86,87,88,89,90,91,92,93,94]. 

With the exception of the Antarctic krill oil [62,63], containing EPA + DHA, which was studied on human, all compounds, reported in Table 1, are in preclinical setting. Indeed, a randomized, double-blind parallel arm trial study, on overweight and obese men and women (*n* = 76) receiving 2 g/day of krill oil, or olive oil for four weeks concludes that krill oil supplementation increases plasma EPA and DHA, and is well tolerated, without adverse effects [62]. The subsequent study showed that 2 g/day of krill oil, for four week, increases the concentration of plasma endocannabinoids in overweight and obese subjects but decreases 2-arachidonoylglycerol (2-AG), only in obese subjects [63]. According to these studies no effects on MetD are produced by krill oil, further researches are warranted. As shown in Table 1, only compounds produced by *Calanus finmarchicus*, *Phorbas* sp., *Aplidium meridianum*, *Undaria pinnati fida*, *Laminaria japonica*, *Cylindrotheca closterium*, and *Hematococcus pluvialis* act specifically on adipose tissue, while other marine drugs target systems beyond the adipose tissue. Drugs from *Axinyssa* sp., *Eurispongia* sp., *Xestospongia testudinaria*, *Hyrtios erectus*, *Penicillium* and *Eurotium* sp. selectively inhibit the Protein tyrosine phosphatase 1B (PTB1B). PTB1B regulates negatively Tyrosine Kinase Receptors-signaling, especially the insulin and leptin receptors [95]. As reported above, low leptin/adiponectin ratios are associated with annual forced expiratory volume in 1 s (FEV1) decline [33]. It has been shown that in women suffering from COPD leptin metabolism is altered with higher secreted leptin levels per BMI strata than in men suffering from COPD [96] It is possible to postulate that leptin secretion increase as well as a gender-dependent dysregulation of adipokine metabolism in patients with COPD compared with BMI-matched controls [97] may contribute to sex differences in COPD pathogenesis through a pathway of chronic systemic inflammation. Women patients may benefit from a treatment that reduces leptin overproduction.

**Figure 5:**
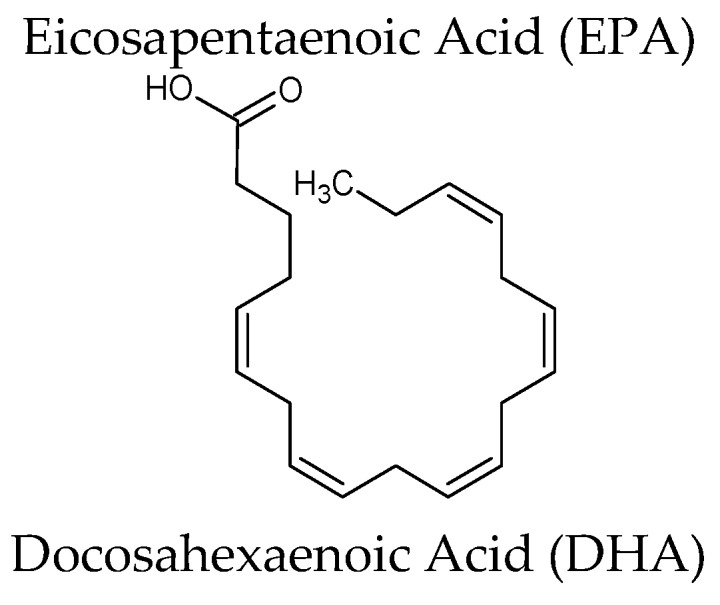

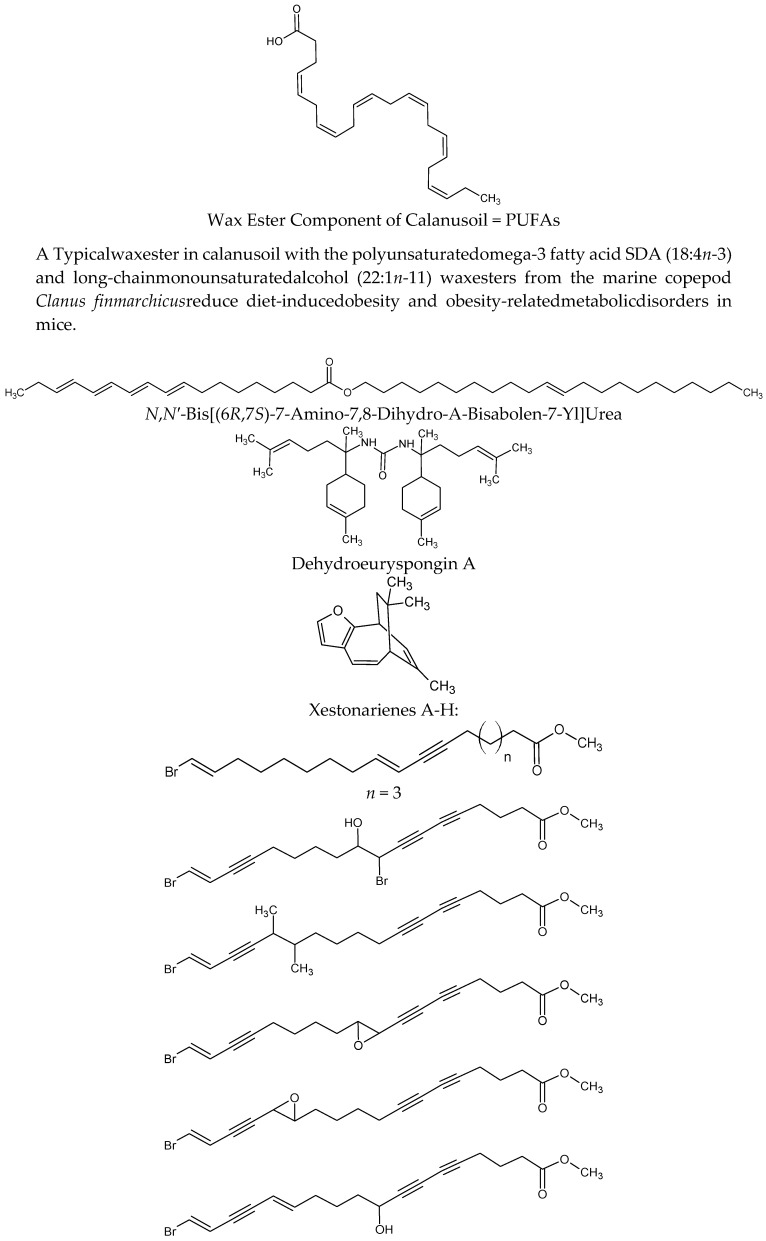

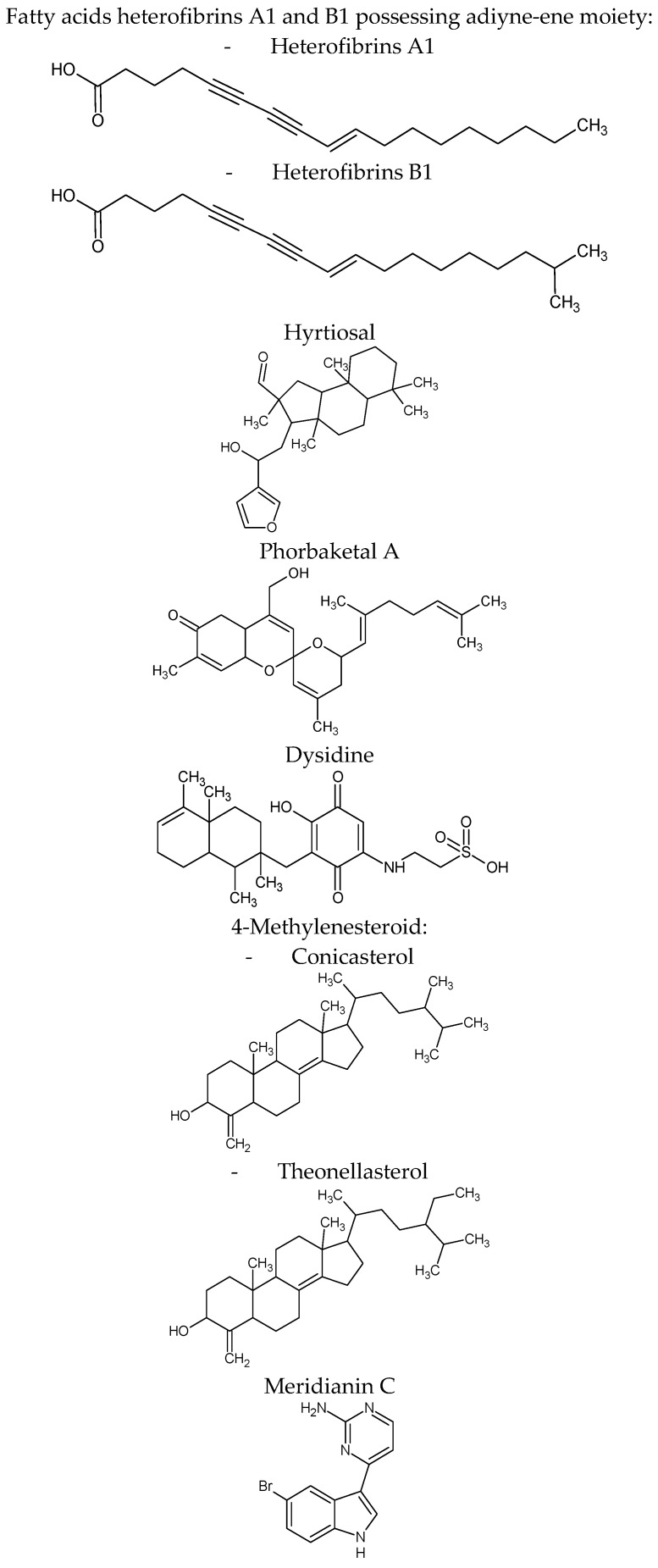

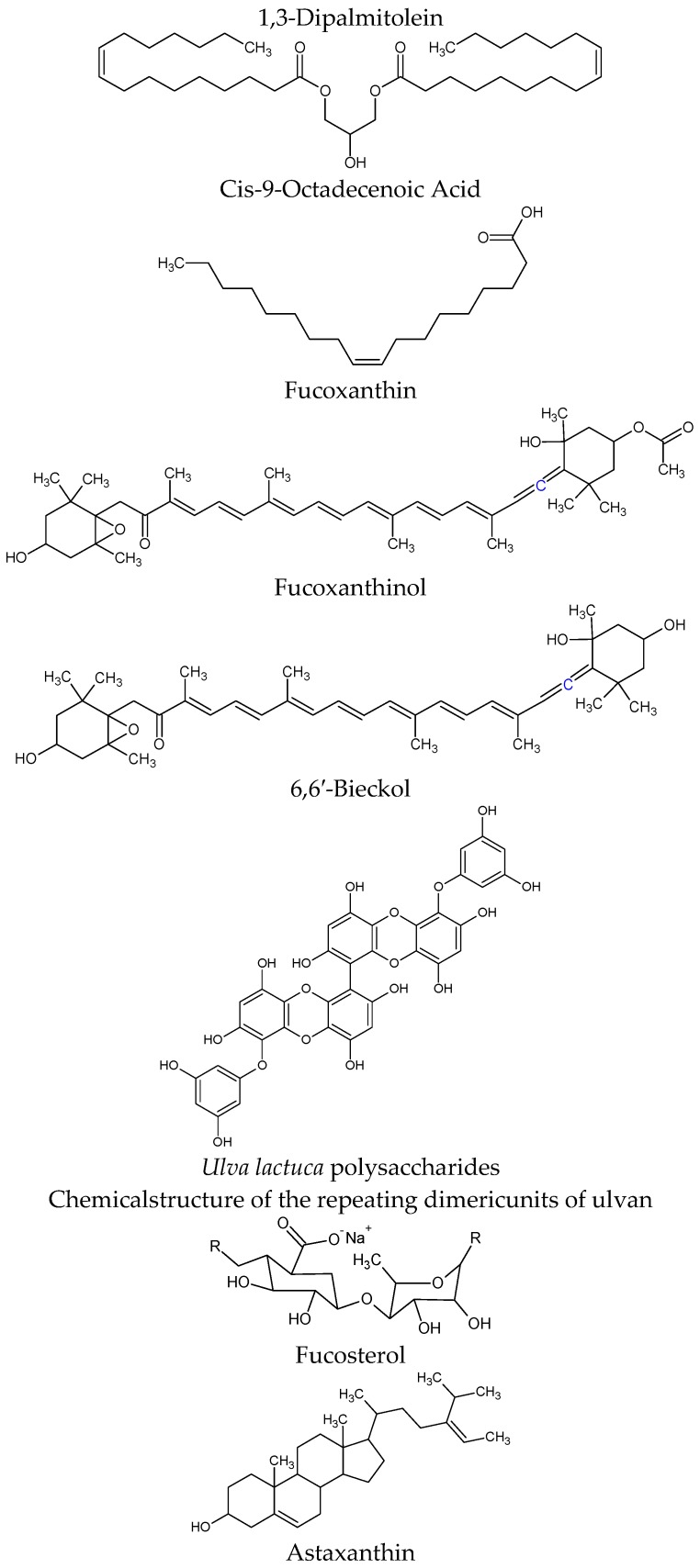

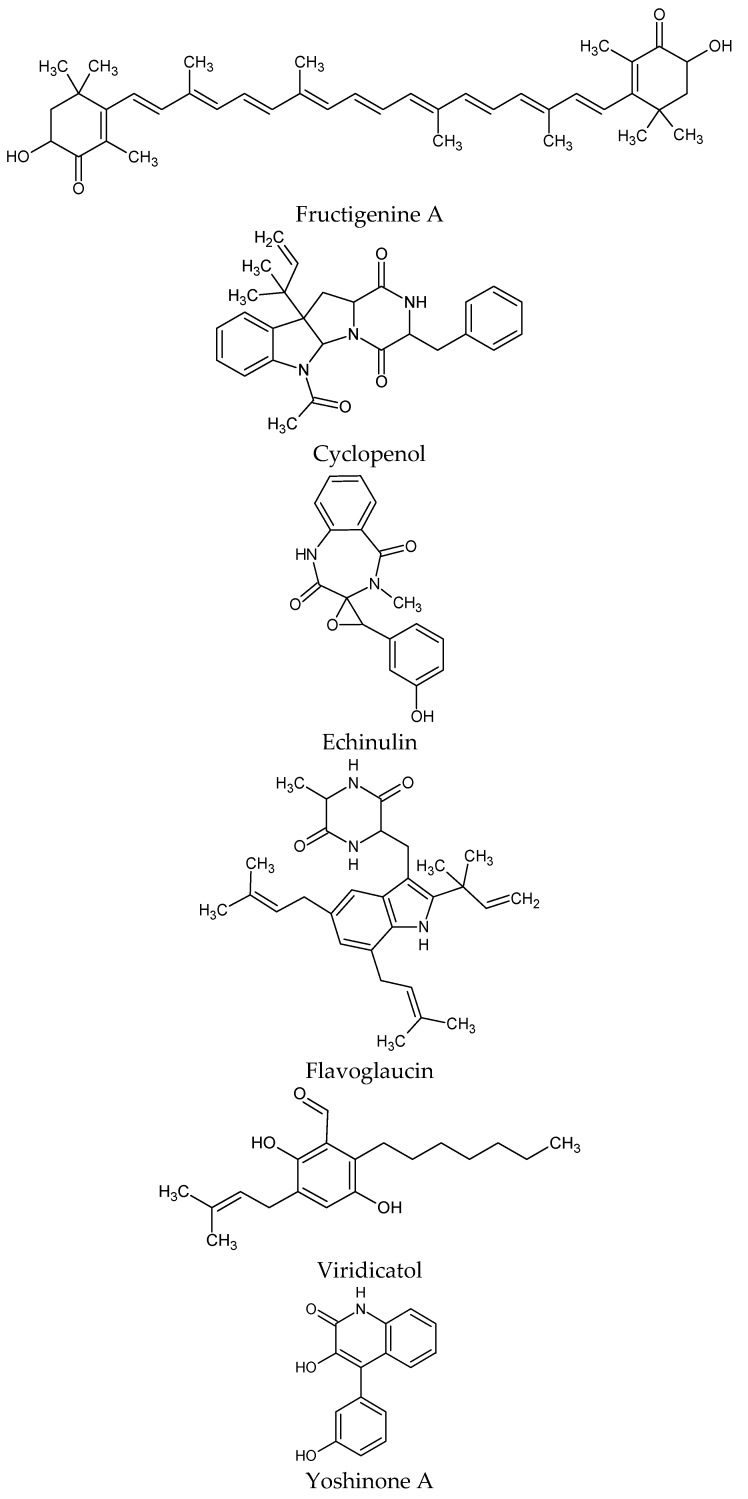

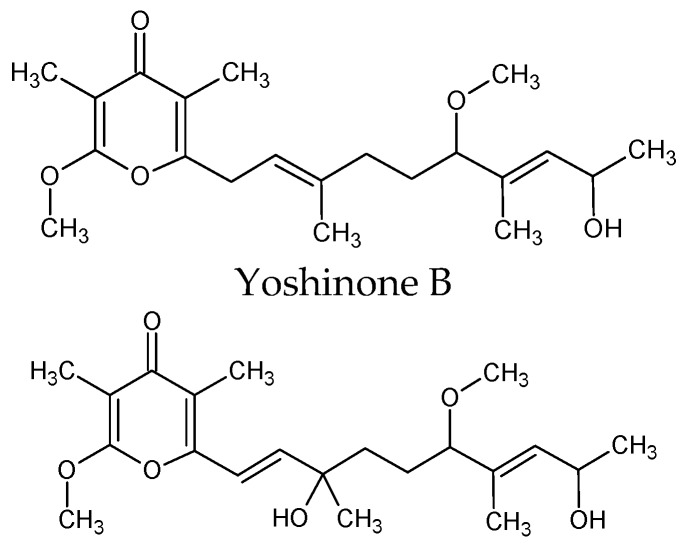
Chemical structures of drugs reported on Table 2.

Figure 5 shows the molecular structures of drugs in Table 2.

## 4. Discussion

Nowadays a single phenotype is determined by interconnected networks involving genome, transcriptome, proteome, metabolome and environment (Figure 3). This vision changes our approaches to disease from simplistic linear approaches to the person’s capacity for health resilience and survival. Consequently, the term “multi-morbidity” viewed as simple sum of single diseases is misleading, reflecting an oversimplification. The so-called “multi-morbidities” are interconnected occurrences among a common interconnected network. In COPD, some co-occurring conditions may be linked to a common mechanism, such as systemic inflammation [3,98]; whether systemic inflammation spreads from airways tract into the circulation or systemic inflammatory state, involving many organs, spreads to the lung remains a question to be determined. Indeed, a study that integrated records from approximately 13 million patients from the Medicare database with disease-gene maps discovered a set of COPD co-morbidity candidate biomarkers that includes IL15, TNF and JUP (junction plakoglobin), and characterizes their association to aging and life-style conditions, such as smoking and physical activity [99]. Obesity and COPD share some mechanisms, such as inflammation, and there is interconnection between pathway activation/deactivation in adipose tissue and lung function modulation. It is well known that indicators of oxidative stress are augmented in COPD and reactive oxygen species (ROS) which may alter signaling pathways and antioxidant molecule function, are implicated in the pathogenesis of COPD, although their role in the development/progression of COPD is not fully proven, as recently reviewed in [100]. On the other hand, high levels of reactive oxygen species (ROS) are strictly linked to obesity and associated pathologies, notably insulin resistance and type 2 diabetes, as reviewed in [101]. It has been reported that ROS generation in contracting skeletal muscle is elevated when there is TNF-α overproduction in the lung and that this can induce muscle dysfunction [102,103] as observed in COPD and obesity.

Since the introduction of the concept of inflammasomes, almost a decade ago, inflammasomes are considered the central player of both innate immune and inflammatory responses [104] and recently are considered implicated in different diseases including metabolic syndrome, obesity, respiratory, cardiovascular and neurodegenerative disorders [104,105,106]. Inflammasomes are complexes of multimeric proteins consisting of a sensor protein, the adapter protein ASC (apoptosis-associated speck-like protein containing a caspase recruitment domain), and caspase-1 [107,108]. The inflammasomes sensor proteins belong either to the NOD-like receptor (NLR) or to the AIM2-like receptor family. NLRP3 initiates pro-inflammatory signaling through recruitment and clustering of ASC, the zymogen protease, and caspase-1. Then caspase-1 is activated triggering a form of cell death called “*pyroptosis*” [107]. NLRP3 inflammasome is implicated in the progression of different non-communicable diseases. Surplus levels in nutrients associated with caloric overload as well as low-level of circulatory endotoxemia (LPS), as in the case of obesity, cause NLRP3 activation playing an important role in the perpetuation of insulin resistance and inflammation [109]. High expression of the NLRP3 genes is observed in the abdominal subcutaneous adipose tissue in obese adolescents [110] as well as in COPD patients [111]. In the neutrophils of COPD patients during acute exacerbation, an upregulation of NLRP3 mRNA expression in comparison with stable disease has been observed [112]. EPA + DHA can abolish the NLRP3 inflammasome activation, inhibiting the subsequent activation of caspase-1 [113].

In this review, different observations supporting our principal hypothesis of a link between obesity and COPD are reported and discussed. Although there is no a direct causal relation, the new biological data, presented here, as well as the clinical and epidemiological observations, reported in literature recently reviewed in [114] tend to suggest a link. Marine drugs acting on adipose tissue or on mechanism of inflammation may be helpful, not only in obesity control but also in subjects with high BMI and concomitant COPD. Whether these treatments affect COPD in a long-term positive perspective requires further investigations.

## Figures and Tables

**Table 1:** FDA/EMA drugs approved obtained by marine sources.

Drug	Systematic (IUPAC) Name	Indication/Mechanism
**Cytarabine [Cytosar-U^®^]** ATC code: L01BC01 Source: *Cryptothecacrypta Phylum:* Bryophyta Class:Bryopsida	4-amino-1-[(2*R*,3*S*,4*R*,5*R*)-3,4-dihydroxy-5-(hydroxymethyl)oxolan-2-yl] pyrimidin-2-one [C_9_H_13_N_3_O_5_]	Anticancer DNA synthesis interference
**Eribulin [Halaven^®^]** ATC code: L01XX41 Synthetic macrocyclic analogue of halichondrin B Source: *Halichondria okadai Phylum:* Porifera Class: Demospongiae	2-(3-Amino-2-hydroxypropyl)hexacosahydro-3-methoxy-26-methyl-20,27-bis(methylene)11,15-18,21-24,28-triepoxy-7,9-ethano-12,15-methano-9*H*,15*H*-furo(3,2-i)furo(2′,3′-5,6) pyrano(4,3-b)(1,4)dioxacyclopentacosin-5-(4*H*)-one [C_40_H_59_NO_11_]	Anticancer Microtubule dynamics inhibitor
**Trabectedin [Yondelis^®^]** ATC code: L01CX01 Source: *Ecteinascidia turbinate Phylum:* Chordata Class: Ascidiacea	(1′*R*,6*R*,6a*R*,7*R*,13*S*,14*S*,16*R*)-6′,8,14-trihydroxy-7′,9-dimethoxy-4,10,23-trimethyl-19-oxo-3′,4′,6,7,12,13,14,16-octahydrospiro[6,16-(epithiopropano-oxymethano)-7,13-imino-6a*H*-1,3-dioxolo[7,8]isoquino[3,2-b][3]benzazocine-20,1′(2′*H*)-isoquinolin]-5-yl acetate [C_39_H_43_N_3_O_11_S]	Anticancer DNA binding and alkylation at the N2 position of G causing DNA bending toward the major groove. Interfering activated transcription, transcription-coupled nucleotide excision repair (TCR) complex poisoning, RNA polymerase degradation, DNA double-strand breaks generation
**Brentuximab [Adcetris^®^]** ATC code: L01XC12 synthetic dolastatin 10 Source: *Dolabella auricularia Phylum*: Mollusca Class: Gastropoda	Antibody-monomethyl auristatin Econjugate [C_6476_H_9930_N_1690_O_2030_S_40_ (C_68_H_105_N_11_O_15_)3–5]	Anticancer Tubulin polymerizationblock
**Ziconotide [Prialt^®^]** ATC code: N02BG08 Source: *Conus magus. Phylum*: Mollusca Class: Gastropoda	Peptide: H-Cys-Lys-Gly-Lys-Gly-Ala-Lys-Cys-Ser-Arg-Leu-Met-Tyr-Asp-Cys-Cys-Thr-Gly-Ser-Cys-Arg-Ser-Gly-Lys-Cys-NH_2_ [C_102_H_172_N_36_O_32_S_7_]	Anti-pain Selective N-type voltage-gated calcium channel blocker
**Vidarabine [Vira-A^®^]** ATC code: J05AB03 Source: *Tectitethya crypta Phylum:* Porifera Class: Demospongiae	(2*R*,3*S*,4*S*,5*R*)-2-(6-amino-9*H*-purin-9-yl)-5-(hydroxymethyl)oxolane-3,4-diol hydrate [C_10_H_15_N_5_O_5_]	Antiviral Viral DNA polymerase inhibitor/substrate
**iota-Carrageenan [Carragelose^®^]** Source: *Eucheuma denticulatum Phylum*: Rhodophyta Class: Florideophyceae	A family of linear sulfated polysaccharides	Antiviral
**Omega-3[Lovaza^®^]** Source: oil of several fish sources	Omega-3-acid ethyl esters (ethyl esters of eicosapentaenoic acid (EPA) and docosahexaenoic acid (DHA)) EPA ethyl ester: [C_22_H_34_O_2_] DHA ethyl ester: [C_24_H_36_O_2_]	Hypertriglyceridemia Adjunct to diet to reduce triglyceride (TG) levels in adult patients with severe (≥500 mg/dL) hypertriglyceridemia (HTG). Increased breakdown of fatty acids; inhibition of diglyceride acyltransferase which is involved in biosynthesis of triglycerides in the liver; and increased activity of lipoprotein lipase in blood

**Table 2:** Marine organisms and drug derivatives for anti-obesity treatment.

**CRUSTACEAN** *Phylum*: Arthropoda			
**Source**	**Drug**	**Target and Activity**	**Reference**
***Euphausia superb***	Eicosapentaenoic acid (EPA) & Docosahexaenoic acid (DHA) (krill oil)	Randomized, double-blind parallel arm trial, overweight and obese men and women (*n* = 76) were randomly assigned to receive double-blind capsules containing 2 g/day of krill oil, menhaden oil, or control (olive) oil for 4 weeks. Plasma EPA and DHA concentrations increased significantly more in the krill oil groups than in the control group.Well tolerated, with no indication of adverse effects on safety parameters. Reduced body weight gain, abdominal fat, and liver triacylglycerol on diet-induced obese mice	[62,63]
***Calanus finmarchicus***	Wax ester component of *Calanus* oil = PUFAs	On diet-induced obesity and obesity-related disorders in mice. C57BL/6J mice fed a high-fat diet (HFD, 45% energy from fat) reduced body-weight gain, abdominal fat accumulation and hepatic steatosis and improved glucose tolerance Calanus oil supplementation reduced adipocyte size and increased the mRNA expression of adiponectin in adipose tissue. It also reduced macrophage infiltration accompanied by reduced mRNA expression of pro-inflammatory cytokines (TNF-α, IL-6 and monocyte chemotactic protein-1)	[64,65]
**SPONGES** *Phylum*: Porifera			
**Source**	**Drug**	**Target and Activity**	**Reference**
***Axinyssa* sp.**	*N*,*N*′-bis[(6*R*,7*S*)-7-amino-7,8-dihydro-α-bisabolen-7-yl]urea	Protein tyrosine phosphatase 1B (PTP1B) inhibitor. Enhances the insulin-stimulated phosphorylation levels of Akt in Huh-7 human hepatoma cells	[66]
***Euryspongia* sp.**	Dehydroeuryspongin A	Protein tyrosine phosphatase 1B (PTP1B) inhibition at IC_50_ = 3.58 μM	[67]
***Xestospongia testudinaria***	Xestonarienes A-HNew steroidal ketone with an ergosta-22,25-diene side chain	Pancreatic lipase (PL) inhibition IC_50_ = 3.11 μM. Decrease in the plasma triglyceride level following an oral lipid challenge in C57BLKS/J male mice	[68,69]
		Protein tyrosine phosphatase 1B (PTP1B) inhibition IC_50_ value = 4.27 ± 0.55 μM	[70]
***Heterofibria***	Fatty acids heterofibrins A1 & B1 possessing a diyne-ene moiety	Lipid droplet formation inhibition in A431 fibroblast cell lines	
***Hyrtios erectus***	Hyrtiosal	Protein tyrosine phosphatase 1B (PTP1B) inhibition with an IC_50_ = 42 μM in a noncompetitive inhibition mode enhances the membrane translocation of the key glucose transporter Glut4 in PTP1B-overexpressed CHO cells facilitate insulin inhibition of Smad2 activation through the PI3K/AKT pathway	[71]
***Phorbas* sp.**	Phorbaketal A (tricyclic sesterterpenoid)	Adipogenic differentiation inhibition as indicated by less fat droplets and decreased expression of adipogenic marker genes. The expression of TAZ (transcriptional coactivator with PDZ-binding motif Phorbaketal A increased the interaction of TAZ and PPARγ to suppress PPARγ (peroxisome proliferator-activated receptor γ) target gene expression	[72]
		Inhibits the production of inflammatory mediators via down-regulation of the of nuclear factor-kappaB (NF-κB), pathway and up-regulation of the heme oxygenase-1 (HO-1) system in LPS-stimulated RAW 264.7 macrophage cells	[73]
***Dysidea villosa***	Dysidine (sesquiterpene quinine)	Differentiated 3T3-L1 cells and resulted in the increased deposition of Glucose transporter type 4 (GLUT4) in the cellular membrane	[74]
***Theonella* sp.**	4-methylenesteroid derivativesconicasteroland heonellasterol)	Pregnane-X-receptor (PXR) modulators PXR is a gene involved in the bilirubin, bile acids, glucose and lipids metabolism	[75]
**TUNICATES** *Phylum*: Chordata (*sub-Phylum:* Tunicata)			
**Source**	**Drug**	**Target and Activity**	**Reference**
***Aplidium meridianum***	Meridianin C derivatives (indole alkaloids)	Inhibition lipid accumulation during 3T3-L1 pre-adipocyte differentiation and lowered leptin expression it influences important differentiation pathways as C/EBP-α, PPARγ and fatty acid synthase	[76]
**ECHINODERM** *Phylum:* Echinodermata			
**Source**	**Drug**	**Target and Activity**	**Reference**
***Stichopus japonicas***	1,3-Dipalmitolein & cis-9-octadecenoic acid	α-Glucosidase inhibitors in Saccharomyces cerevisiae IC_50_ = 4.45 and 14.87 μM	[77]
**ALGAE** *Phylum***:** Euglenozoa			
**Source**	**Drug**	**Target and Activity**	**Reference**
***Undaria pinnati fida*, *Laminaria japonica* (macroalgae, brown seaweeds) & *Cylindrotheca closterium* (microalgae)**	Fucoxanthin	Induces uncoupling protein 1 (UCP1) in abdominal white adipose tissue (WAT) mitochondria, leading to the oxidation of fatty acids and heat production in WAT regulation of cytokine secretions from both abdominal adipose cells and macrophage cells infiltrated into adipose tissue	[78,79]
		Regulates mRNA expression of inflammatory adipocytokines involved in insulin resistance, iNOS, and COX-2 in WAT and has specific effects on diabetic/obese KK-A(y) mice, but not on lean C57BL/6J mice	[80,81]
		Inhibits lipase activity in the gastrointestinal lumen and suppress triglyceride absorption, and fucoxanthin was converted to fucoxanthinol in the intestine and released into the lymph in conscious rats	[82]
		Fucoxanthin upregulates the expression of uncoupling protein 1 (UCP1) and adipokine mRNA in white adipose tissue (WAT) of diabetic/obese KK-A(y) mice	[83]
		Down-regulates SCD1 expression and alters fatty acid composition of the liver via regulation of leptin signaling in hyperleptinemia KK-A(y) mice but not in leptin-deficient ob/ob mice	[84]
***Ecklonia stolonifera* (brown algae)**	Fucoxanthinol/Fucoxanthin Metabolite	3T3-L1 adipocyte cells and a RAW264.7 macrophage cell co-culture system. A diet containing 0.1% Fx was fed to diabetic model KK-Ay mice for three weeks Fx diet significantly improved glucose tolerance compared with the control diet group.In in vitro studies, FxOH showed suppressed tumor necrosis factor-α (TNF-α), and monocyte chemotactic protein-1 (MCP-1) mRNA expression and protein levels in a co-culture of adipocyte and macrophage cells	[85]
		Inhibits expression of PPARγ and C/EBPα, resulting in a decrease of lipid accumulation in 3T3-L1 pre-adipocytes,3T3-L1 pre-adipocytes differentiation	[86]
**ALGAE** *Phylum***:** Euglenozoa			
**Source**	**Drug**	**Target and Activity**	**Reference**
***Eisenia bicyclis* (brown algae)**	6,6′-bieckol	Decreased lipid accumulation and expression levels of peroxisome proliferator-activated receptor γ (PPARγ), CCATT/enhancer-binding protein α (C/EBPα) and sterol regulatory element binding protein-1c (SREBP-1c) (mRNA and protein), and fatty acid synthase and acyl-coA carboxylase (mRNA). inhibition of differentiation of 3T3-L1 adipocytes	[87]
***Ulva lactuca***	*Ulva lactuca* polysaccharides (ULPS)	α-amylase and maltase inhibition leading to a significant decrease in blood glucose rate	[88]
***Phaeodactylum tricornutum***	Fucosterol	C57BL/6 mice a high-fat diet supplemented with PT powder (15% or 30% *w*/*v*) for 12 weeks, and determined energy intake, weight loss, and lipid profiles each week reduced body weight gain, and epididymal and perirenal adipose tissue weight via activation of AMPK and HMGCR pathways	[89]
***Hematococcus pluvialis***	Astaxanthin	Male Swiss albino mice: starch-based control diet or a high fat-high fructose diet (HFFD). Fifteen days later, mice in each dietary group were divided into two and were treated with either ASX (6 mg·kg^−1^ per day) in olive oil or olive oil alone. For 60 days ASX treatment reduced lipid levels and oxidative stress in skeletal muscle and adipose tissue and improved insulin signaling by enhancing the autophosphorylation of insulin receptor-β (IR-β), IRS-1 associated PI3-kinase step, phospho-Akt/Akt ratio and GLUT-4 translocation in skeletal muscle	[90]
		Pre-treatment with ASTA (10 µM) for 1 h attenuates the LPS-induced toxicity and ROS production. In U937 cells stimulated with LPS (10 µg/mL)	[91]
		Astaxanthin inhibited the increases in body weight and weight of adipose tissue that result from feeding mice a high-fat diet, reduced liver weight, liver triglyceride, plasma triglyceride, and total cholesterol	[92]
**FUNGI** *Phylum:* Ascomycota			
**Source**	**Drug**	**Target and Activity**	**Reference**
***Penicillium* spp. *Eurotium* sp.**	Fructigenine A Cyclopenol Echinulin Flavoglaucin Viridicatol	Selective inhibition of PTP1B fructigenine A in a noncompetitive manner, viridicatol in a competitive manner	[93]
**CYANOBACTERIA** *Phylum*: Cyanobacteria			
**Source**	**Drug**	**Target and activity**	**Reference**
***Leptolyngbya* sp.**	γ-pyrones yoshinone	Osteoclastogenesis, protein kinase inhibitor. Inhibitory activity against the adipogenic differentiation of 3T3-L1 cells IC_50_ = 420 nM. Mice at high-fat diet (HFD) for 5 weeks received kalkipyrone at a dosage of 5 mg·kg^−1^/day showed effective suppression of adipose tissue weight gain in mice	[94]
